# Supplementary material for: Phytochemical Investigation of Myrcianthes cisplatensis: Structural Characterization of New p-Coumaroyl Alkylphloroglucinols and Antimicrobial Evaluation against Staphylococcus aureus
Source: Plants (Basel). 2023 Feb 24;12(5):1046. doi: 10.3390/plants12051046 (PMC10005737; doi:10.3390/plants12051046)

Figure S1: NOESY experiment of compound **1**.

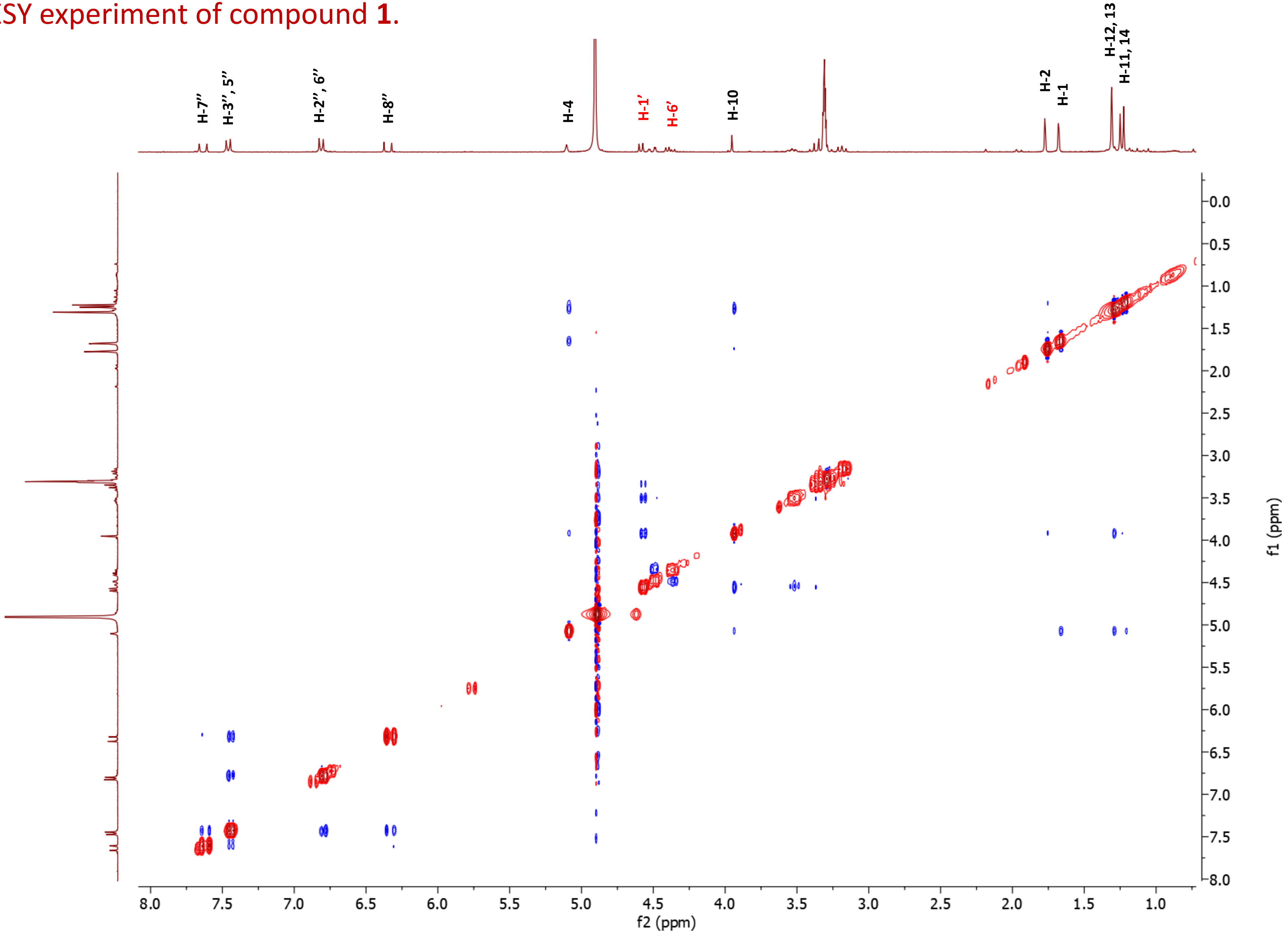

Figure S2: HMBC experiment of compound **3**.

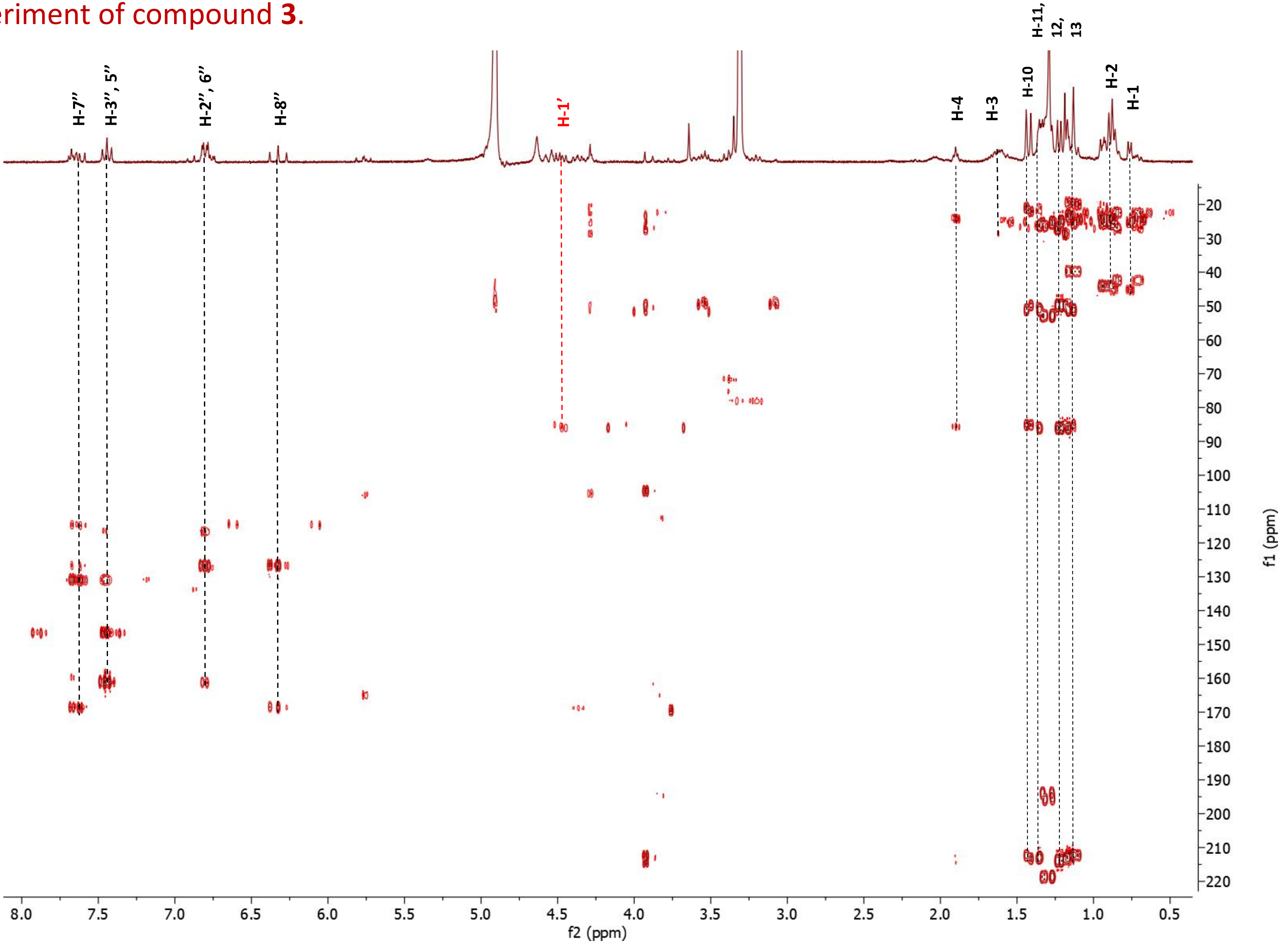

Figure S3, Panel A: 2D-NMR elucidation of MYR_A: HSQC experiment.

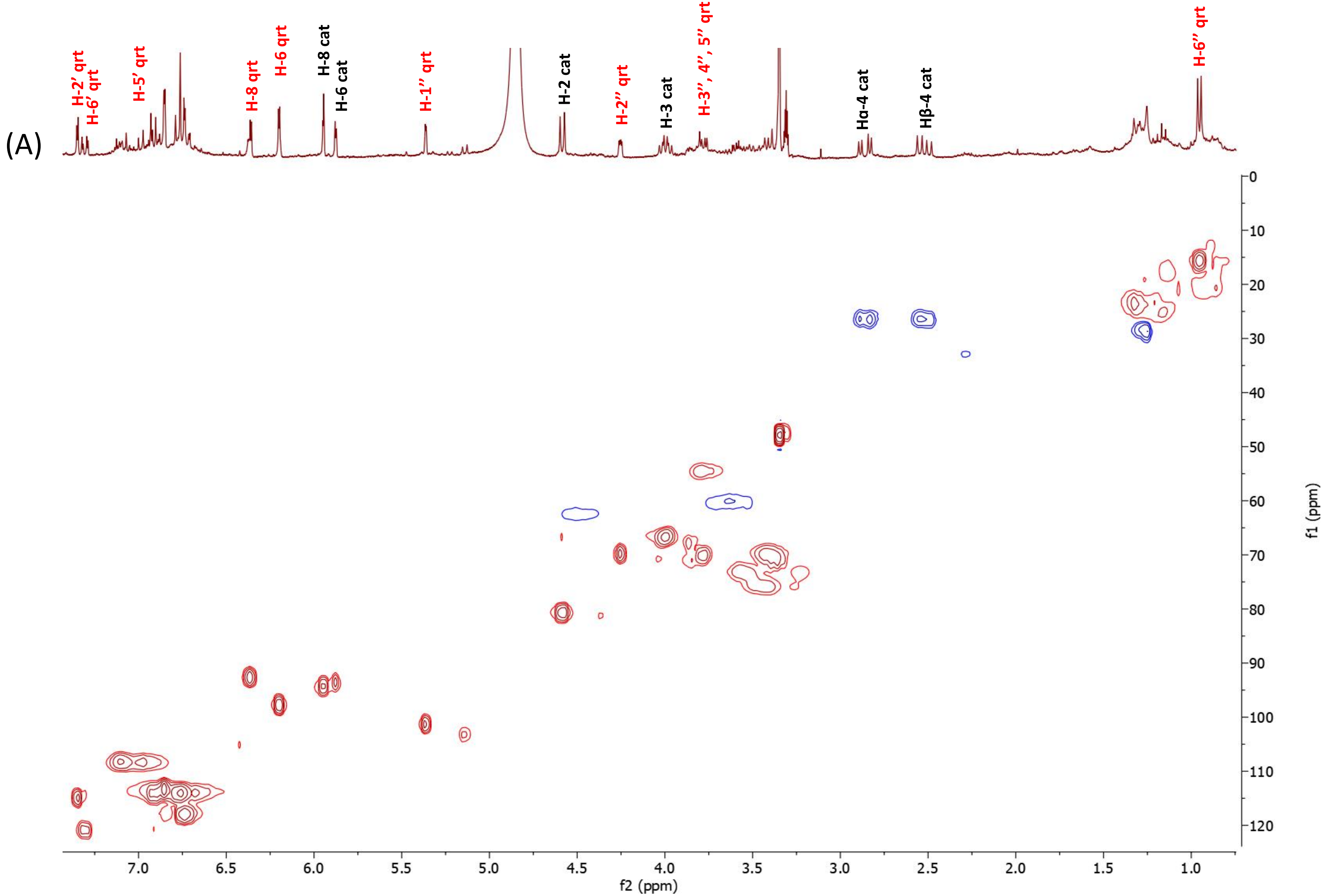

Figure S3, Panel B: 2D-NMR elucidation of MYR_A: HMBC experiment.

(B)

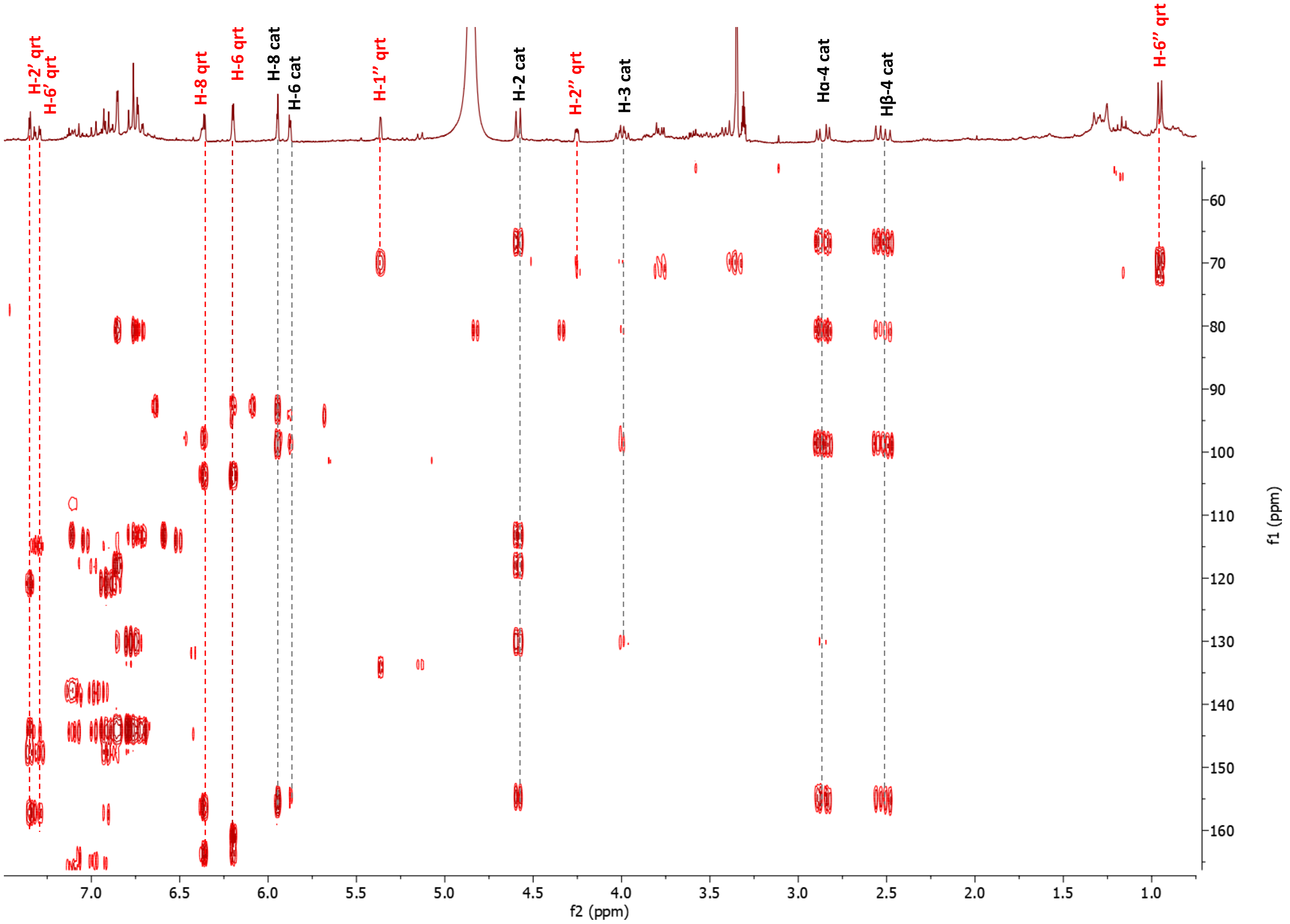

Supplement: Supplementary file 1 [file plants-12-01046-s001.zip › plants-2206761-supplementary.pdf]
